# How Does the Execution of the Pilates Method and Therapeutic Exercise Influence Back Pain and Postural Alignment in Children Who Play String Instruments? A Randomized Controlled Pilot Study

**DOI:** 10.3390/ijerph17207436

**Published:** 2020-10-13

**Authors:** Carolina Poncela-Skupien, Elena Pinero-Pinto, Carmen Martínez-Cepa, Juan Carlos Zuil-Escobar, Rita Pilar Romero-Galisteo, Rocío Palomo-Carrión

**Affiliations:** 1 Integral Rehabilitation Center, CRI. 38001 Santa Cruz de Tenerife, Spain; carolponsku@gmail.com; 2 Department of Physical Therapy, Faculty of Nursery, Physiotherapy and Podiatry, University of Seville, 41004 Seville, Spain; 3 Department of Physiotherapy, Faculty of Medicine, CEU-San Pablo University, 28003 Madrid, Spain; jczuil@ceu.es; 4 Department of Physiotherapy, Faculty of Science Health, University of Málaga, 29016 Málaga, Spain; rpromero@uma.es; 5 Department of Nursery, Physiotherapy and Occupational Therapy, Faculty of Physiotherapy and Nursery, University of Castilla-La Mancha, 45071 Toledo, Spain; Rocio.Palomo@uclm.es; 6 GIFTO. Physiotherapy Research Group of Toledo, 45071 Toledo, Spain

**Keywords:** child, exercises, pilates-based, exercise therapy, musculoskeletal pain, spine, string instruments

## Abstract

*Background*: Inappropriate posture in children while playing some string instruments can cause back pain and alterations of the spine. To date, there is no research on the effect of exercise on children who play a musical instrument, although it is known that transversus abdominis muscle control through the Pilates method has shown pain reduction and posture improvement in this population. *Objective*: To assess the effectiveness of the Pilates method combined with therapeutic exercise with respect to therapeutic exercise exclusively in reducing pain and improving postural alignment in children playing string instruments applying a protocol of low dose to increase children’s adherence to training. *Methods*: A randomized controlled pilot study was designed with two parallel intervention groups. Twenty-five children (10–14 years old) were randomized in two intervention groups: Pilates method with therapeutic exercise (experimental) and therapeutic exercise (control) for 4 weeks (50 min per day, one day per week). Two assessments were performed (before and after treatment) to assess back pain and shoulders and hips alignment using a visual analog scale and the Kinovea program. *Results*: Statistically significant differences were obtained for pain reduction before (*p* = 0.04) and after (*p* = 0.01) playing the instrument in the experimental group. There were no significant changes in alignment improvement in any of the two groups. *Conclusion*: The application of a low dose of the Pilates method combined with therapeutic exercise could be a beneficial intervention for pain reduction before and after musical practice in children who play string instruments.

## 1. Introduction

To produce a harmonic sound, musicians must adopt a specific posture and find the gesture that is most suitable to be able to play the instrument. The musical gesture can produce various conditions that can be approached from different perspectives or health specialties [1].

Playing a musical instrument is a highly complex motor skill. The regular daily practice, rehearsals, and performances generate a training load that places a great demand on the musculoskeletal system [2]. Perfecting the musical gesture and progressing in the acquisition of skills requires many hours of practice, which involves intensive training [3]. This daily practice implies a high physical stress, which makes musicians susceptible to musculoskeletal injuries [4] and pain [5]. In a similar way to athletes, certain injuries are more frequently related to the practice of certain instruments. For example, shoulder injuries are more common in musicians who play the violin or viola [6,7]. These injuries usually result from muscle overuse and poor postural habits, often due to the lack of required physical fitness [8]. Moreover, chronic pain is a serious problem in orchestra musicians, due to very specific playing techniques and body positions adopted while playing, with a high impact on the professional and personal life [9]. This playing-related pain has been associated with an increase in hours of practice [10].

Within the wide variety of musical instruments, string instruments stand out in terms of difficult posture, especially rubbed string instruments: violin, viola, cello, and double bass. Musicians playing string instruments have a higher risk of musculoskeletal injuries, with a percentage between 65% and 88% [11,12]. The leading causes of these musculoskeletal problems in violinists and violists are overuse, compression of the nerves, and focal dystonia, which impede the musician to play the instrument correctly [13]. Incorrect posture, along with repetitive movements in musicians playing string instruments, can lead to the onset of thoracic outlet syndrome, which presents pain in the neck, shoulder and arm. In this way, the appearance of injuries could be prevented or improved [14,15,16]. In addition, shoulder and back position affect expiratory capacity; specifically, a straight back and extrarotated shoulders can increase resistance to the air passage in the superior airway tract and affect the amount of air that can enter the lungs [17].

Furthermore, like any practice-dependent activity or the acquisition of any skill, playing a musical instrument requires focused attention and self-discipline, prioritizing practice over other more instantly gratifying activities. This is particularly important in a group setting, where there are many social distractors, such as side conversations or a piece being played incorrectly by others, which musicians must ignore for the benefit of the performance [18]. Despite this, children aged 10–11 years, who were musically trained, have shown reduced reaction time differences between congruent and incongruent trials with respect to children without musical training [19].

In this sense, musicians’ advantages in inhibition skills may relate to paralleled processes in disciplined instrumental practice; learning to play a musical instrument involves frequent stopping to correct mistakes and rehearsing small passages in isolation, which both delay the reward of playing a piece in its entirety and require one to put aside immediate non-musical distractors for the larger reward of musical proficiency. These skills may additionally relate to musicians’ regular practice of error monitoring, where children playing music must quickly adjust their motor behavior in response to unanticipated musical demands and mistakes [20]. During the first four years of musical training, the child acquires a series of skills in the posture and handling of the instrument. However, the greatest ability occurs after the fifth year of practice, where errors in practice are reduced and the posture begins to be more structured and with little variability, which could lead to alterations in the alignment structured after that year and, subsequently, chronic pain [20,21].

Such age, i.e., early adolescence, could be considered as the period between 10 and 14 years of age. It is at this stage when physical changes usually begin to manifest with a sudden growth spurt [22]. Internal changes also take place, thus it is the best time for them to learn about awareness of their own body and even improving their body image [23,24]. Thus, children aged 10 to 14 years who play string instruments, while the spine continues to consolidate, an incorrect position at school, while sitting in the classroom, together with an improper position or technique during the practice of the violin, viola, cello, or double bass, can cause scoliotic postures that affect children playing these instruments [25,26]. Thus, authors such as González- Gálvez et al. [27] highlight the positive effects of physical exercise on the aspects mentioned above. Regarding exercise, Taylor et al. [28] defined therapeutic exercise as a physical activity program in which the patient performs a voluntary muscular contraction and/or body movement to alleviate symptoms, improve function or improve, maintain, or delay health deterioration. Musicians must be in good physical condition in order to be able to play their instruments, since a certain position with the instrument must be kept during rehearsals. Therefore, it is necessary to have a good technique to reduce possible injuries [1]. The factors to consider when reducing the possibility of musculoskeletal injuries are: complex musculoskeletal skills, frequency of repetitive movements, the influence of static and dynamic musculature, posture, technique, and hours spent practicing with the instrument [1]. Many musicians focus on practicing with the instrument, leaving out other important aspects, such as the continuous performance of physical activity. Resistance training in professional musicians shows a strengthening in the extensor muscles of the back, arm, and wrist. It also shows an improvement in mobility and performance with the instrument [29]. Musculoskeletal conditions affect the back, neck, shoulder, knee, and many other parts of the body, and they can result in reduced function and a poorer quality of life for individuals, as well as in increased demands on health services [30]. The persistent nature of musculoskeletal pain may have a physical, psychological, and social impact on the person, leading to sedentary lifestyles [31].

Another form of physical exercise is the practice of Pilates. This method, created by Joseph Hubertus Pilates at the beginning of the 20th century, is a combination of different specialties, such as yoga, gymnastics, and traumatology, combining muscular strength with mental, respiratory, and relaxation control. The method focuses on the development of internal muscles to maintain body balance and give stability and firmness to the spine, making it one of the most widely used methods as rehabilitation therapy, as well as to prevent and improve back pain [32]. The exercises of this method can help to strengthen the abdominal muscles in both genders, improving the activation and control of the transversus abdominis muscle, flexibility, endurance, and trunk muscle activity compared to people who do not exercise [33]. This suggests that individuals can improve their muscular endurance, flexibility, and reduce the possible age injuries, using relatively low-intensity Pilates exercises that do not require equipment or a high skill and are easy to master and perform within a personal fitness routine [34,35]. Its benefits have been demonstrated in young people and adolescents and the exercises are suitable for all ages, all body types, and all fitness abilities, due to the modifiable nature of the movements [36,37,38,39].

It is well known that adolescence is a critical period for sagittal disposition, as several longitudinal studies have found that there is an increase in thoracic kyphosis (especially in men), lumbar lordosis (especially in women), and anterior pelvic tilt during this developmental stage, although some cross-sectional studies have found that the changes during childhood and adolescence could depend on sex and anthropometric variables. As a result, the percentage of misalignment increases during the childhood and adolescence stages [39,40]. Reduced flexibility in childhood and adolescence can lead to an increase in the risk of cervical tension in adulthood and a higher prevalence of lumbar hyperlordosis [40]. Thus, exercise programs that include stretching can promote a significant increase in flexibility in school age children and regular physical activity leads to improvements in the health of children and adolescents [41,42].

However, during the musicians´ learning process, their physical condition or postural habits necessary to prevent injuries associated with instrument practice are not frequently considered [8]. Therefore, it was decided to incorporate the Pilates method in this study in combination with therapeutic exercise, since the literature affirms that Pilates exercise combines core stability, posture, flexibility, strength, breathing, and movement control, including different exercises with different levels of intensity; moreover, this method produces positive benefits, including a reduction in pain and disability, thus improving the physical and emotional components of quality of life [27,39,41,43,44].

There are several application protocols for the Pilates method with different dosage: 12 weeks of treatment with a frequency of one hour per week in children with type 1 diabetes mellitus [45], 9 months with two 15-min sessions per week aimed at adolescents to reduce sagittal curvature and improve hamstring extensibility [27], 4 weeks with a frequency of 5 one-hour sessions per week aimed at improving the body composition of adolescent girls [46]. All these protocols have produced good results in the variables measured during the execution time. Thus, an easy-to-follow protocol for groups was created for the present study, where the participants’ motivation was encouraged due to their age, the training skill that could be acquired in the near future, and the fact that it was to be completed at home. A low dose protocol with 4 weeks was established, using one session per week, since it combined the Pilates method and therapeutic exercise, which suggested that both would produce improvements in a short time. The school environment is an ideal setting for interventions to be applied, since large numbers of children and adolescents can be addressed [47]. With the assumption that motivation is the key to initiate and sustain beneficial health behaviors, theory-based intervention studies apply motivational strategies to increase children’s participation in physical activity. Amotivation can be described as a state of passiveness, i.e., a lack of intentionality and motivation to act, which can be based on a feeling of lack of competence or arise from a lack of interest, relevance, or value in a given action [46]. To increase the motivation for and adherence to physical activity, it is important to reduce the time of the exercise and prolong the total dose [48]. Thus, the aim of the present study was to determine whether executing one 50-min session per week for 4 weeks allowed children to acquire adherence and commitment to the application of the exercises, with the aim of applying a protocol with a group session guided by the therapist, as well as the execution of a daily session at home. Consequently, training them to incorporate physical activity into their routine would contribute to preventing chronic pain and non-modifiable musculoskeletal alignment.

To the best of our knowledge, this is the first study to evaluate how the execution of the Pilates method and therapeutic exercise influences back pain and postural alignment in children who play string instruments using a protocol with a low-dose treatment (4 weeks/50 min per week).The objective of this study was to assess the effectiveness of the Pilates method combined with therapeutic exercise compared to therapeutic exercise alone in reducing pain and improving postural alignment in children (10–14 years old) who play string instruments applying a low-dose protocol.

## 2. Materials and Methods

### 2.1. Study Design

A single-blind (evaluator) randomized controlled pilot study was designed (with clinical.gov registry number NCT04438707) using two parallel intervention groups. Convenience sampling was used to access the population of individuals who met the selection criteria and who belonged to the two music schools (Barrios Orquestados) where the study was carried out. The randomization was conducted at the music schools, for which an external blinded person chose an envelope with the therapy to be performed in the music schools. The study was approved by the San Pablo CEU University ethics committee of Madrid (270–20-TFM) according to the World Medical Association Declaration of Helsinki. 

### 2.2. Participants

The sample was recruited from two musical schools of Barrios Orquestados (Conservatory of Music) in Tenerife (Spain). Children from these two schools and their parents were provided with the information on the objectives and procedures to be carried out in the project.

The study included children with typical development who did not have cognitive or motor alterations or different pathologies that could affect the postural motor control and the interaction of the child with his/her environment [49], aged between 10 and 14 years, who played the violin, viola, cello, or double bass, or who learned to play the violin, viola, cello, or double bass in the current school year or up to a maximum of four previous years. The study excluded children with pathologies related to structured musculoskeletal alterations (bone diseases, neuromuscular diseases, lesions of the central or peripheral nervous system, presence of scoliosis, kyphosis or alignment alterations that could not be modified), affectations that directly influence the correction of posture or pain due to an organic or systemic cause, low effort tolerance (interpreted as a fundamental failure of the stress system after a period of severe or prolonged physical and/or psychosocial stress in vulnerable individuals such as children who do not practice physical activity) [50], and an insufficient cognitive level to understand the basic exercises.

Once the informed consent was signed by the parents or legal guardians, the children could participate in the study.

### 2.3. Interventions

Two intervention groups were created: a group that performed the Pilates method combined with therapeutic exercise and a control group in which only therapeutic exercise was carried out. Both groups conducted their corresponding intervention programs for 4 weeks with a frequency of one therapy class per week, which lasted 50 min. This protocol is based on short-term exercises for less than 6 weeks, with a frequency of 1 or 2 times per week and an average duration of 45 min per session. The execution of a protocol of these characteristics aimed at adolescents will promote adherence and continuation after treatment as a therapeutic activity [38].

Prolonged standing or sitting postures while practicing with the instrument, such as the violin, viola, double bass, or cello, result in inadequate body and instrument positioning [9].

**Figure 1:**
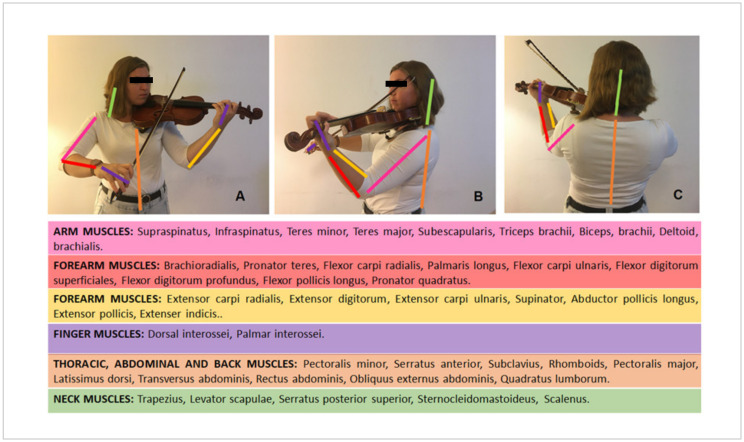
Representation of different muscles involved when the violin is played. Picture (**A**) shows an anterior view of the position when the violin is played and the involved muscles, picture (**B**) represents a lateral view, and picture (**C**) shows a posterior view of the same action.

The area that tends to be overexposed in these positions is the lumbar area, which is why it is common for children to suffer from back pain [13]. It is believed that the time spent in an inadequate seated position can be considered a risk factor for the development of postural changes in the sagittal plane and back pain [51]. These results are alarming in view of the level of impairment and loss of quality of life which musicians suffer as a consequence of chronic pain [9]. Musicians must be motivated to address their health problems openly and practice physical therapy at an early stage [52]. When a string instrument is played, various muscles are activated depending on the adopted position required. Figure 1 shows the musculature involved when the violin instrument is played. 

The classes were taught by a professional physical therapist in both methods, who previously instructed the children on the execution of the exercises and the postures they had to acquire to execute the proposed exercises adequately and without complications. In the 4 weeks of treatment, each class was divided into different periods for both groups: warm-up (10 min), training (30 min), and stretching (10 min) (Table 1). 

**Figure 2:**
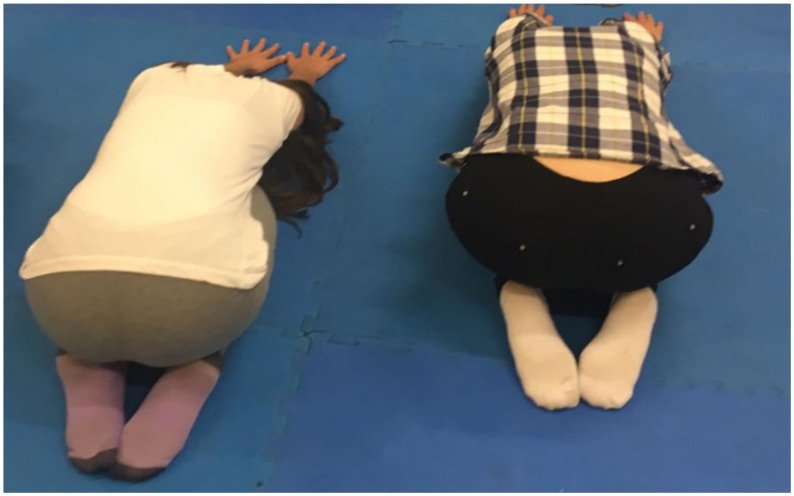
Stretching period for both intervention groups.

The first and last periods of each class were the same in both groups (Figure 2). 

**Figure 3:**
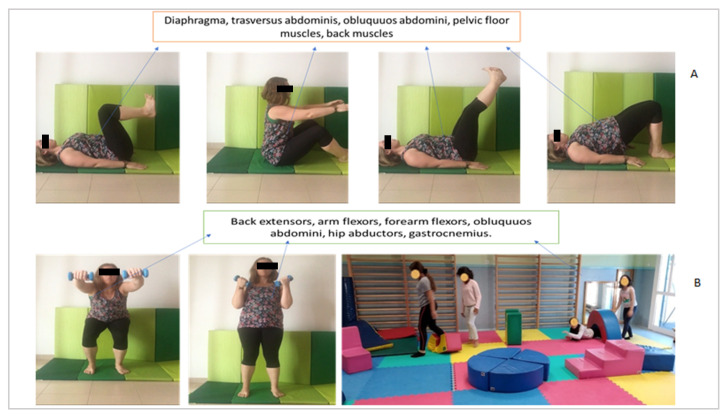
Pilates method and therapeutic exercise training with the involved muscles. Part (**A**) shows the Pilates exercises, each exercise is performed for five minutes with 3 sets of 10 reps each (20 min of training). For the different exercises, the chin is placed low, the shoulders back and down; the child activates the transverse and pelvic floor. In the first exercise, the legs are kept at 90º; then, maintaining the posture, the child works the whole body globally, focusing on the abdomen and legs. In the second exercise, the child rolls down to the sacrum and focuses on the abdomen. In the third exercise, the child works the whole body globally, focusing on the abdomen and legs. Part (**B**) shows the application of 10 min of therapeutic exercise: 2 exercises with dumbbells (5 min) with 15 reps for each exercise, finishing the training with 5 min of an exercise circuit combining actions such as jumping, dragging, running, going up and down the stairs, throwing balls, and other games.

**Figure 4:**
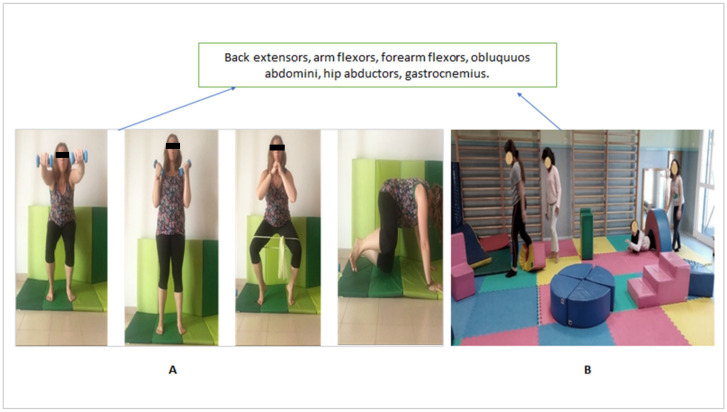
Therapeutic exercise training with the involved muscles: the training lasted 30 min. Part (**A**) is based on the execution of different exercises for the first 20 min: each exercise is performed for 5 min, with 3 10-rep sets. The first exercise consists of performing a squat while raising the arms to shoulder height, and then going back to the starting position (with arms extended along the body and hips aligned with the feet). In the second exercise, a slight flexion of the knees is performed, flexing and extending the elbows with the dumbbells (the starting position would be the same as in exercise 1). In the third exercise, an elastic band is placed right above the knees. The exercise begins with the legs slightly separated; then, the child tiptoes, flexing the knees slightly and separating the hips. The last exercise begins in quadruped position (distributing the weight among both knees and hands), from which the child transfers the weight of the body to one knee in order to raise the other knee to hip height; then, the child returns to the starting position and repeats the exercise with the other knee. Part (**B**) shows the end of the training, which lasts 10 min. This second part consists of an exercise circuit with different ludic movements: races, ball throws, jumps, drags, games in pairs and in groups, global movement of the body with rings or other elements, going up and down steps, ramps, etc.

Figure 3 shows the different exercises performed in the Pilates method and therapeutic exercise training with a time of 30 min: 20 min for the first part and 10 min for the therapeutic exercise. In the Figure 4, can be observed the exercises sequence in the therapeutic exercise group.

Video S1 shows the different parts of the Pilates method combined with therapeutic exercise and the therapeutic exercise alone.

### 2.4. Assessments, Study Variables, and Measuring Instruments

**Figure 5:**
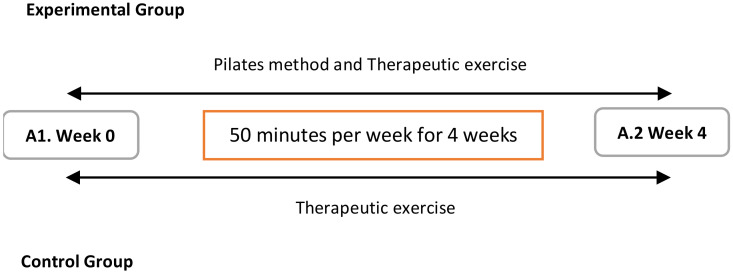
Measurement times. A1. Week 0: Assessment 1: Baseline situation pre-treatment; A2. Week 4: Assessment 2: Post-treatment results after 4 weeks of intervention for experimental and control group.

A blind therapist performed two assessments of the study variables: one before the intervention (week 0), to establish the baseline situation, and another one after the intervention (week 4), to analyze the changes after the application of the protocols (Figure 5).

#### 2.4.1. Study Variables and Measuring Instruments

##### First Outcome Measurement: Back Pain Perceived before, during, and after Practicing with the String Instrument

The tool chosen for pain measurement was the visual analog scale (VAS) [53,54]. It consists of a 0 to 10 cm horizontal line, at the ends of which are the extreme expressions of a symptom. On the left side, the absence or lesser intensity is located, while the right side represents the greater intensity. The participants place a mark on the line that best describes their pain, which is then measured with a millimeter ruler. The intensity is expressed in centimeters or millimeter. Since it has more than 10 intensity units, it allows greater detail of the pain rating. The scale value reliably reflects the intensity of pain and its evolution. Therefore, it serves to assess how pain intensity evolves in different people [54].

A questionnaire with the following three questions was used to analyze the sensation of pain: Does your back hurt before playing the instrument? If yes, how much does it hurt from 0 (least painful) to 10 (most painful)? Does your back hurt while playing the instrument? If yes, how much does it hurt from 0 (least painful) to 10 (most painful)? Does your back hurt after playing the instrument? If yes, how much does it hurt from 0 (least painful) to 10 (most painful)?

##### Second Outcome Measurement: Postural Alignment: Shoulders and Hips

The Kinovea software was used to measure the alignment of the shoulders and hips. Kinovea is an open-source video analysis program that allows observing videos and making traces and annotations at specific moments, to later select fragments and save them independently. Moreover, it has a wide variety of analyses and measurement tools to add descriptions, draw lines, and calculate distances. This program also offers a dual screen-based comparison system. In this way, it is possible to check how the same person, or 2 different people, perform an exercise. This will make it very easy to observe defects and improvements in execution. Then, the data can be saved in spreadsheets. The study carried out by Puig-Diví et al. [55] indicates that Kinovea is a reliable and valid program at distances of up to 5 m with an angle of 90º to 45º.

**Figure 6:**
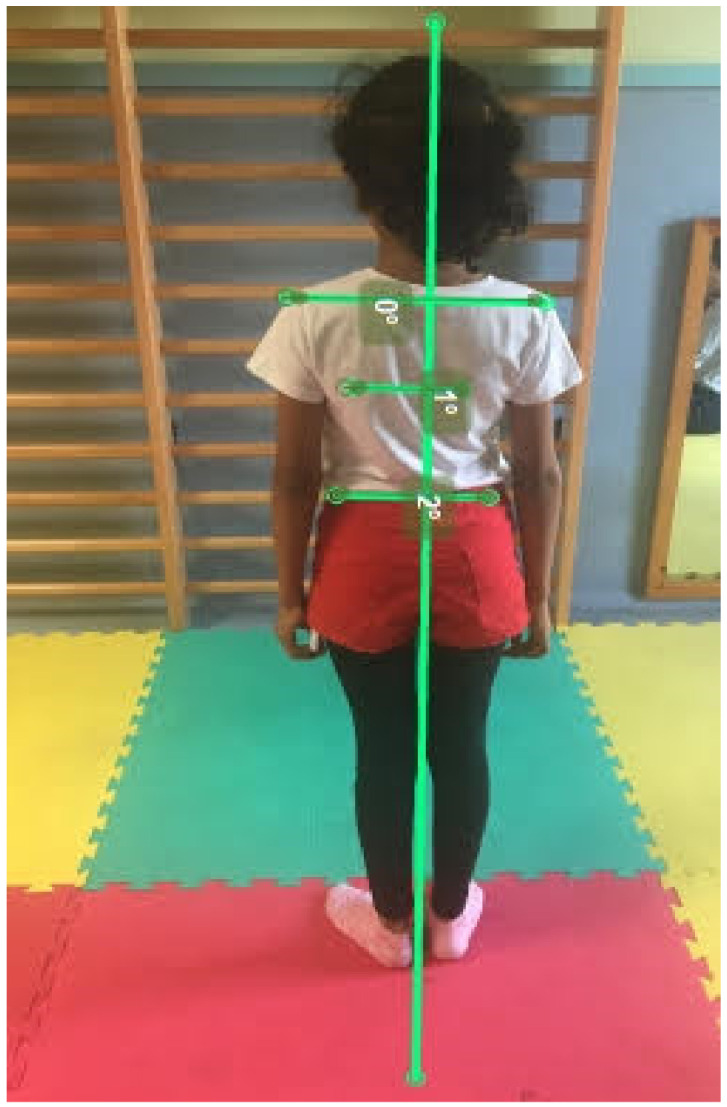
Back capture and representation of the horizontal tracings between the bone structures and the vertical line for the calculation of the degrees in the measurements through the Kinovea software.

A photo of the children’s back was taken. Subsequently, Kinovea calculated the degrees of alignment. To this end, two horizontal lines were drawn: one at the level of the shoulders and another one at the level of the hips (the measurements were taken in the frontal plane). A vertical line was drawn down the middle of the body, in order to divide it into two halves. The degrees of difference were obtained using the horizontal and vertical lines (Figure 6). For example, if one shoulder was higher than the other due to a slight scoliosis, a 5º difference could be observed between one shoulder and the other.

### 2.5. Statistical Analysis

An intention-to-treat analysis was performed. Statistical analyses were performed using SPSS 20.0 (SPSS Inc., Chicago, IL, USA). Given the small sample size, we used non-parametric tests. Fisher’s exact test was used to determine inter-group differences according to sex. The group differences were studied with the Mann–Whitney U–test, using Wilcoxon signed-rank tests for pre-post comparisons in each group, expressed in medians (IQR: interquartile range). Significance was set at a *p*-value of 0.05 (*p* values expressed with Bonferroni correction). The Spearman’s correlation test was used to measure the correlation of the post–pre-intervention difference in the value of back pain (before-during-after playing the instrument) with age and time of practice.

## 3. Results

**Figure 7:**
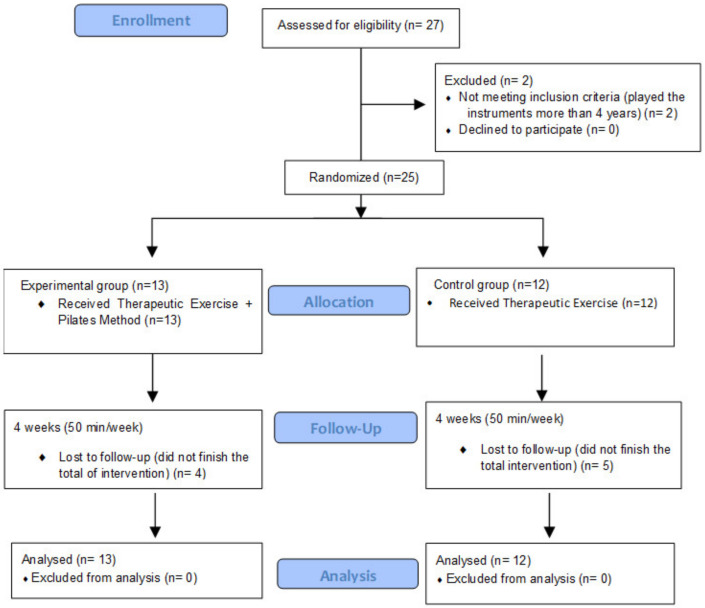
Consort flow diagram of progress through the phases of the randomized clinical trial of the therapeutic exercise–Pilates method and therapeutic exercise groups (enrollment, allocation, intervention, follow-up, and analysis of the participants).

Twenty-seven participants were contacted and screened for participation in the pilot study. Two (7.4%) participants were not enrolled, since both had played a string instrument for over 4 years. Twenty-five children were randomized: 13 in the experimental group (8 females [61.54%] and 5 males [38.46%]) and 12 in the control group (9 females [75%] and 3 males [25%]) (Figure 7).

The average age was 13 years (SD = 1.6) for the experimental group and 11.17 years (SD = 0.72) for the control group. The time in months that they had played the instrument was an average of 23.53 (SD = 11.66) in the experimental group and 34 (SD = 16.84) in the control group. During the follow-up, 4 and 5 subjects were lost (due to illness, change of residence, and abandonment of music school) in the experimental and control groups, respectively, whereas 9 and 7 children completed the treatment in the experimental and control groups, respectively (Table 2). 

There were no statistically significant differences between the control and experimental groups at baseline with respect to sex (*p* = 0.08), time playing the string instrument (*p* = 0.6), time spent playing the instrument per week (*p* = 1), years of experience playing the instruments in the music school (*p* = 0.6), pain (before-during and after playing the instrument) and shoulders and hips alignment (*p* > 0.05) (Table 2). However, there were statistically significant differences at baseline in terms of age, with children in the control group beginning to play the instrument at a slightly younger age (*p* < 0.01 and *p* = 0.04, respectively in the Mann–Whitney U-test).

### 3.1. Primary Outcomes: Pain Perceived before, during, and after Practicing with the String Instrument

**Figure 8:**
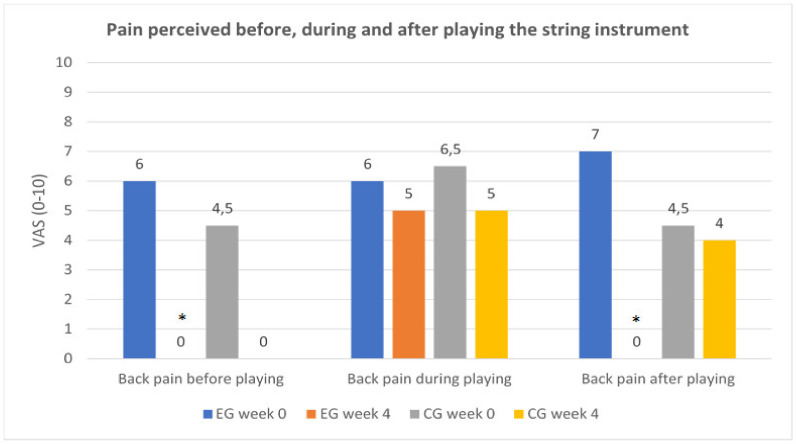
Progression of back pain before, during, and after playing the instrument in the EG (experimental group: Pilates method combined with therapeutic exercise) and CG (control group: therapeutic exercise) in the basal situation (week 0) and after treatment (week 4). VAS: visual analog scale (score: 0–10); *: statistically significant differences (*p* < 0.05) between week 0-week 4.

In the experimental group (Pilates method and therapeutic exercise), significant changes were obtained in the back pain assessment before playing (*p* = 0.04) and after playing the instrument (*p* = 0.01) (Figure 8), but not during playing (*p* = 0.24). In the control group (therapeutic exercise), no significant changes were obtained in back pain before, during, or after playing the instrument (*p* > 0.05). The greatest decrease was obtained in back pain before playing for the control group and in back pain before and after playing the instrument for the experimental group (Table 3).

### 3.2. Secondary Outcomes

The shoulder and hips alignment showed no significant changes in any of the two groups (shoulder alignment in the experimental group: *p* = 0.09; shoulder alignment in the control group: *p* = 0.70; hips alignment in the experimental group: *p* = 0.57; hips alignment in the control group: *p* = 0.10) (Table 4).

### 3.3. Third Outcomes: Correlation of the Experimental Group Post–Pre-Treatment Difference in Back Pain (Pre–During–After) with Age and Time Playing the Instrument

Correlation was only observed for the experimental group between age and post–pre-treatment difference in back pain before playing the instrument (r = −5.9), (Table 5). No correlation between variables were observed for the control group.

## 4. Discussion

This study evaluated the effectiveness of the Pilates method combined with therapeutic exercise compared to therapeutic exercise alone in reducing pain and improving postural alignment in children (10–14 years) who play string instruments. The-low dose protocol applied proved to be effective in reducing pain. 

Practicing with a musical instrument requires a large number of hours maintaining a particular posture that causes pain and alteration of the postural alignment [20]. If this condition is maintained over time, it could lead to scoliosis, back pain, and general postural asymmetry, which would limit the quality of life of young musicians [19]. In addition to the postures maintained by musicians and excessive muscular activity, there are other risk factors that can cause pain, such as the hours of muscle training, the type of instrument played, and the weight of the instrument [56]. The International Conference of Symphony and Opera Musicians survey reported a high frequency of musculoskeletal problems in string players (60%); considering the prevalence of pain according to the type of instrument played, 68% of musculoskeletal pain in the upper limbs and back have been reported in string musicians [57]. Thus, it is important to start a motor and posture control at an early stage to prevent these injuries, including the Pilates method, since it reduces pain, as is shown in our study in the experimental group, with improvements in back pain before and after playing the string instrument. 

In addition, the children in the present study played string instruments 4 h per week (both groups), which can influence back pain and alignment. As was demonstrated in the study of Kaufman-Cohen and Ratzon, performed in orchestra musicians, eight hours playing the instrument per week are correlated with upper limb pain [56]. These findings suggest that the time spent playing the instrument is a risk factor to consider in order to prevent pain. In the present study, the Pilates method combined with therapeutic exercise allowed children to reduce back pain, since no changes were detected in the therapeutic exercise applied alone [56]. Another risk factor is the weight of the instrument, which has been studied in adult orchestra musicians, showing a correlation with the upper limbs pain and physical functions [56]. This correlation could also be in adolescents and produce back pain and more posture alterations due to periods of rapid growth, since the body is very susceptible to physical stress. Considering that the peak onset of low back pain is between 12 and 14 years of age, it appears that the onset of low back pain is related to the growth spurt in children, when the rapidly growing spine is sensitive to excessive loads, in addition to the incorrect postures that children with this age hold for long periods of time when playing heavy instruments [58]. If they start with postural reeducation within the therapeutic activity, such as the Pilates method combined with therapeutic exercise, this protocol could reduce pain before and after playing the instrument, as shown in this study, with statistically significant changes. 

There are no studies on the effectiveness of both intervention methods in children who play musical instruments. Herein lies the novelty of this study compared to other published studies. The effects of exercise in general have been analyzed, although, to the best of our knowledge, the joint intervention of Pilates and therapeutic exercise has not been analyzed before in children who play musical instruments. Therefore, we compared the data related to pain and postural control with that of other studies that have addressed these variables.

After analyzing the obtained results with respect to back pain in the two study groups, it is suggested that the application of the Pilates method has a fundamental role in reducing such pain, since statistically significant changes were only observed in the group that combines the Pilates method with therapeutic exercise. Neither the experimental group nor the control group perceived pain before playing the instrument at 4 weeks of training, although this perception of no pain was only maintained in the experimental group after playing the instrument when the intervention finished, while the control group only reduced the perception of pain after treatment in 0.5 points. The changes in pain during the time spent playing the instrument were hardly reduced for both groups, probably due to the position held during the practice with the instrument and the weight of the latter. Such correlation between the pain perceived by the musician during the musical practice and the weight of the instrument was demonstrated in the study of Kaufman-Cohen and Ratzon [56]. After playing the instrument, the children of the experimental group (Pilates method combined with therapeutic exercise) did not perceive pain, which can be attributed to motor control and the activation of the appropriate musculature to better adapt to the demands of the instrument, such as the transversus abdominis. This would further stabilize the pelvis and lumbar spine, thus generating more fluid movements and improving motor behavior [42].

Our results are in agreement with those reported by Rydeard et al. [22]. In their study, patients participated in specific Pilates training focused on the mechanisms of neuromuscular control, which was effective in reducing pain and improving muscle function. Such improvement could have been due to the involvement of the transversus abdominis muscle, lumbar multifidus, diaphragm, and pelvic floor, in addition to the stabilization of the gluteus maximus muscles. These findings are also in line with those reported by Ludvig [59], who emphasized the importance of the transversus abdominis muscle in stabilizing the spine during exercises of the abdominal wall in order to reduce pain. Improvements in motor control and pain reduction have been reported in different populations with the application of the Pilates method using different doses: in the study of Mendonça et al. [60] to treat juvenile idiopathic arthritis using 6 months of treatment, and in the study of Alves de Araújo et al. [42] performed in women with non-structural scoliosis with 3 months of treatment. Improvements have also been found in a short-time protocol like the one applied in the present study, using 6 weeks of Pilates in postmenopausal women, with great results in pain reduction [61]. These findings would be based on the activation of the transversus abdominis (since it is one of the fundamental principles of Pilates) in the experimental group, who performed the Pilates method and therapeutic exercise, suggesting that this could have been an important factor in reducing the pain before and after the instrument practice, as different studies point out [10,62]. 

Our results are not in line with those of Cibinello et al. [41], who concluded that Pilates has not been confirmed to have an effect on the posterior chain flexibility and trunk mobility in healthy school-aged children. The children who underwent the intervention program of 28 sessions of exercises based on Pilates showed improvements in flexibility and mobility test results; however, there were no differences between these children and those of the control group. Nevertheless, it must be taken into account that the intensity of the intervention and the population is different from those of our study. The characteristics of the participants may be a factor to consider in obtaining the results after applying the training.

In the present study, both groups of children play string instruments (violin, viola, cello, and double bass) in a similar percentage, which is a factor that could produce pain before playing the instrument, along with the position adopted by adolescent musicians. However, improvements were only found before playing the instrument in the experimental group compared with the control group. Therefore, during the adolescent growth spurt, specific stabilizing and stretching exercises could promote better muscular stabilization mechanisms and correct alignment of the spine [58]. Due to the incorrect postural exposure and the age at which young musicians suffer pain, the Pilates method combined with therapeutic exercise could be beneficial in reducing pain, improving posture, and increasing adherence to the instrument practice [63].

The execution of a 4-week protocol with 50 min per day, one day per week, would be effective in reducing back pain (before-after playing the string instrument) in the combination of the Pilates method with therapeutic exercise, since improvements were obtained with respect to the therapeutic exercise group. However, no significant changes were observed in the postural improvement and alignment of the shoulders and hips, since, despite the improvement in the experimental group (Pilates method and therapeutic exercise), the changes were not statistically significant, suggesting that the control of the transversus abdominis muscle could be enough to reduce pain, although it may be necessary to further automate postural control in order to obtain a noticeable improvement in the postural alignment of the shoulders and hips, in addition to scapular control, which is also a principle of the Pilates method [64]. In addition, it could improve pain, since the exercises would effectively recruit the transversus abdominis muscle, internal oblique, diaphragm, and pelvic floor, thus preventing the participation of accessory muscles, such as the hamstrings, posterior and anterior cervical muscles and lumbar muscles [65]. It would be appropriate to study the transversus abdominis muscle activation and its influence on reducing back pain, measured by electromyography [66]. 

Another factor to bear in mind in postural control could have been baseline alignment, since children in both groups had a low deviation in the alignment. In this sense, children with a worse condition or a greater alteration in postural alignment may influence the clinical changes obtained in these variables. Thus, the study of Schreiber et al. [67] assessed the effect of six months of specific Schroth exercises (a type of therapeutic exercise) for scoliosis (spinal asymmetry) combined with standard care versus standard care without exercises, obtaining greater results in reducing the severity of the school-curves when performing specific exercises. This could indicate that the baseline situation, as well as the training dose, could interfere with the results. Likewise, it was inferred that the application of therapeutic exercise, as well as the combination of the Pilates method and therapeutic exercise, could have a long-term effect on alignment (scapulae), although more than 4 weeks of intervention would be necessary.

Since this was a pilot study to determine changes in pain and alignment of shoulders and hips after the intervention, it was decided to carry out two assessments (baseline: week 0, and post-treatment: week 4). In this way, it could be observed whether the intervention methods applied in the two groups had a short-term effect on the study population. No relevant changes in alignment were obtained with this protocol for the measures of the variables. These findings are in line with those reported by Kuo et al. [68], who only found small improvements in thoracic kyphosis during standing. Despite our results, other investigations have obtained significant results that support the effectiveness of Pilates as a method to correct posture [27,42,69], among which there is a systematic review of studies conducted in children and youth [70]. Therefore, in a future study, we would consider the possibility of increasing the dose while maintaining the same total duration (4 weeks). Due to the adherence of the children in both groups, and their motivation in the execution of the exercises, as well as the acquisition of the capacity to self-train at home, it could be proposed to execute a 4-week protocol, 50 min per day, 5 days a week, in which a group session such as the one carried out in the present study would be allocated to encourage motivation and adherence and make the necessary corrections, thus avoiding complications, and 4 sessions at home carried out by the child previously trained in the physical activity. A follow-up of the results would be performed at 3 and 6 months post-treatment; i.e., 4 time points would be considered in a future investigation (week 0, week 4, week 16, and week 28). Jago et al. [71] concluded in their research that the participants, girls between the ages of 10 and 12, enjoyed Pilates for 4 weeks and attended regularly, suggesting that Pilates could be a useful means of increasing physical activity in young people. Pilates can be a useful method to increase physical activity, and thus it deserves further research. The relatively low cost of delivering Pilates sessions (space, instructor, and mats) also indicates that it is an activity that could be implemented within preventive programs in musicians, suggesting that it could be widely disseminated.

There was a moderate correlation (r = 0.59) between age and the post–pre-intervention difference in back pain before playing the instrument for experimental group. This finding suggests that older children can obtain greater control in the execution of the imposed exercises, as well as better understand their execution and, therefore, would obtain greater benefits than younger children. Some studies [72,73,74] show that adults can adapt to moderate or high intensity exercise and feel satisfied with their performance. However, for adolescents, it would be more pleasant to perform a low- or moderate-intensity exercise like the one shown in the present study, since the time intervals are shorter and would allow a better adaptation to its practice and follow-up [72,73,74]. Children are active in low-intensity activity and would be more motivated to participate in activities that they find reinforcing [75]. Motor skills can be learned explicitly and implicitly. Explicit learning makes use of declarative knowledge to build up a set of performance rules that guide motor performance or skills. Individuals are aware of the goal, the rules, and the expected outcome of the task to learn [76,77,78]. Explicit learning depends on age and working memory, suggesting that a better learning would be obtained in older children [78]. Thus, age could influence the intensity executed and also the proposal of exercises, putting more interest in a good practice to obtain benefits after exercise in older children than in younger children, for whom the exercise should be more playful for its correct execution and whose work memory has not fully developed yet [79,80]. These findings from previous studies were also observed in the present study, since all the children completed the training sessions with great interest and motivation, and they stated their satisfaction after the execution of both protocols and their intention to continue with the practice. 

There was no correlation for any of the two groups between the post–pre-intervention difference in back pain (before–during–after) playing the instrument and time spent playing the instrument, due to the fact that the time of experience playing the instrument and the time spent weekly may have not been intense enough for there to be a correlation between the two variables, since, in the study of Ioannou and Altenmüller [10], musicians over 15 years of age practiced with their instruments for a larger number of hours per week. This increase in the number of hours results from the greater demand to acquire musical skills for the instrument practice, due to the requirement to participate in different events, such as concerts and other musical performances. These findings demonstrate the correlation between the time of musical training and pain in adolescent musicians.

The limitations of this study were the small sample size and the age differences among the participants, as well as the lack of post-intervention follow-up to observe the evolution of the results. Future studies should include a larger sample of children, with homogeneous ages, and increase the protocol dosing time to determine whether there are significant changes in the alignment of the shoulders and hips to improve the postural control of children who play string instruments. Moreover, to draw broader conclusions about the impact of music training throughout society, further research should involve participants from more diverse backgrounds. Another interesting future line of research could be the study of neuroplasticity and related brain networks in children and adolescents who play music, following the proposition of Hennessy et al. [20]. 

## 5. Conclusions

The application of a low dose of Pilates method combined with therapeutic exercise could be a beneficial intervention in the reduction of pain before and after practice in children who play string instruments. 

## Figures and Tables

**Table 1:** Intervention protocols design.

Groups	Intervention Protocols Examples
**Therapeutic Exercise**	Each 50-minute class was divided into warm-up, training, and stretching: ○ Warm-up: joint mobility exercises, followed by muscle warm-up exercises (10 min). ○ Training: Abdominal, leg, and arm exercises, exercise circuit (30 min). ○ Stretching: global and analytical stretching to assist the participants in their practice with string instruments (10 min).
**Pilates Method and Therapeutic Exercise**	Each 50 minute-class was divided into warm-up, training, and stretching: ○ Warm-up: joint mobility exercises, followed by muscle warm-up exercises (10 min). ○ Training: Pilates exercises of axial elongation or lengthening, rolling the pelvis and shoulders back and down for 20 min These were combined with therapeutic exercise: abdominal, leg, and arm exercises and exercise circuit for 10 min (total dose: 30 min). ○ Stretching: global and analytical stretching to assist the participants in their practice with string instruments (10 min).

**Table 2:** Baseline characteristics of the participants in the experimental and control groups.

	Experimental Group	Control Group	*p*-Value
**Female (%)**	61.54	75	-
**Male (%)**	38.46	25	-
**Age (years), mean (SD)**	13 (1.6)	11.17 (0.72)	< 0.01
**Time playing the instrument(months), mean(SD)**	23.53 (11.66)	34 (16.84)	0.6
**Time spent playing the instrument per week (Monday- Friday), mean expressed in hours (SD)**	4 (0.32)	4 (0.35)	1
**Years of experience playing the instruments in the music school, mean (SD)**	2 (0.24)	3 (0.29)	0.6
**Music initiation age, mean (SD)**	10.2 (0.65)	8.5 (0.53)	0.04
**String instrument played:**			
**Violin/viola n. (%)**	5 (38.46)	4 (33.33)	-
**Cello n. (%)**	4 (30.77)	4 (33.33)	-
**Double bass n. (%)**	4 (30.77)	4 (33.33)	-
**Self-report outcome median (IQR)**			
**VAS (0–10)**			
**-Back pain before playing**	6 (0, 8)	4.50 (0, 9)	0.7
**-Back pain during practice**	6 (0, 8)	6.50 (0, 9)	0.9
**-Back pain after playing**	7 (0, 8)	4.50 (0, 9)	0.6
**Measured outcome**			
**median (IQR)**			
**Kinovea (movement angles, degrees º)**			
**-Shoulders**	2 (0, 6)	1 (0, 5)	0.6
**-Hips**	2 (0, 6)	1 (0, 5)	0.6

Control group: therapeutic exercise; experimental group: Pilates method and therapeutic exercise; VAS: visual analog scale.

**Table 3:** Results of back pain in the experimental and control groups.

Self-Report Outcome Median (IQR) VAS (0–10)	Experimental Group Week 0	Experimental Group Week 4	*p-*Value	Control Group Week 0	Control Group Week 4	*p*-Value
**Back pain before playing**	6 (0,8)	0 (0, 8)	0.04 *	4.50 (0, 9)	0 (0, 7)	0.08
**Back pain during practice**	6 (0, 8)	5 (0, 7)	0.24	6.50 (0,9)	5 (0, 7)	0.34
**Back pain after playing**	7 (0, 8)	0 (0, 7)	0.01 *	4.50 (0,9)	4 (0, 7)	0.46

Control group: therapeutic exercise; experimental group: Pilates method and therapeutic exercise; VAS: visual analog scale; results expressed in median (interquartile range); * statistically significant differences when *p*-value <0.05.

**Table 4:** Results of postural alignment in the experimental and control groups.

Measured Outcome Median (IQR) Kinovea (Movement Angles, Degreesº)	Experimental Group Week 0	Experimental Group Week 4	*p*-Value	Control Group Week 0	Control Group Week 4	*p*-Value
**-Shoulders**	2 (0, 6)	2 (1, 4)	0.09	1 (0, 5)	0 (0, 2)	0.7
**-Hips**	2 (0, 6)	2 (0, 5)	0.57	1 (0, 4)	0 (0, 3)	0.1

Control group: therapeutic exercise; experimental group: Pilates method and therapeutic exercise; results expressed in median (interquartile range); statistically significant differences when *p*-value <0.05.

**Table 5:** Spearman’s rank correlation coefficient. Correlation of the experimental group post–pre-treatment difference in back pain (pre–during–after) with age and time playing the instrument.

Experimental Group’s Post–Pre-Treatment Difference	Age	Time Playing the Instrument
Back pain before playing the instrument	−0.59 *	−0.05
Back pain during playing the instrument	0.04	0.11
Back pain after playing the instrument	−0.35	−0.18

Values expressed in Spearman’s rank (r); * the correlation is significant at the 0.05 level (bilateral).

## Supplementary Materials

The following are available online at https://www.mdpi.com/1660-4601/17/20/7436/s1, Video S1: Execution of the Pilates method combined with therapeutic exercise and therapeutic exercise alone.
